# An App-Based Intervention for Pediatric Weight Management: Pre-Post Acceptability and Feasibility Trial

**DOI:** 10.2196/36837

**Published:** 2023-04-24

**Authors:** Jennifer S Cox, Elanor C Hinton, Julian Hamilton Shield, Natalia S Lawrence

**Affiliations:** 1 NIHR Bristol Biomedical Research Centre Nutrition Theme Bristol United Kingdom; 2 Psychology Department University of Exeter Exeter United Kingdom

**Keywords:** obesity, pediatric, intervention, eHealth, weight management

## Abstract

**Background:**

A multidisciplinary approach to weight management is offered at tier 3 pediatric weight management services in the United Kingdom. Encouraging dietary change is a major aim, with patients meeting with dieticians, endocrinologists, psychologists, nurse specialists, and social workers on average every other month.

**Objective:**

This research sought to trial an inhibitory control training smartphone app—FoodT—with the clinic population of a pediatric weight management service. FoodT has shown positive impacts on food choice in adult users, with resulting weight loss. It was hoped that when delivered as an adjunctive treatment alongside the extensive social, medical, psychological, and dietetic interventions already offered at the clinic, the introduction of inhibitory control training may offer patients another tool that supports eating choice. In this feasibility trial, recruitment, retention, and app use were the primary outcomes. An extensive battery of measures was included to test the feasibility and acceptability of these measures for future powered trials.

**Methods:**

FoodT was offered to pediatric patients and their parents during a routine clinic appointment, and patients were asked to use the app at home every day for the first week and once per week for the rest of the month. Feasibility and acceptability were measured in terms of recruitment, engagement with the app, and retention to the trial. A battery of psychometric tests was given before and after app use to assess the acceptability of collecting data on changes to food choices and experiences that would inform future trial work.

**Results:**

A total of 12 children and 10 parents consented (22/62, 35% of those approached). Further, 1 child and no parents achieved the recommended training schedule. No participants completed the posttrial measures. The reasons for not wanting to be recruited to the trial included participants not considering their weight to be connected to eating choices and not feeling that the app suited their needs. No reasons are known for noncompletion.

**Conclusions:**

It is unclear whether the intervention itself or the research processes, including the battery of measures, prevented completion. It is therefore difficult to make any decisions as to the value that the app has within this setting. Important lessons have been learned from this research that have potential broad relevance, including the importance of co-designing interventions with service users and avoiding deterring people from early-stage participation in extensive data collection.

## Introduction

Children with a BMI of greater than the 99th percentile for their age and sex who are experiencing or are at risk of health complications resulting from their weight and those with safeguarding concerns may be referred to a tier 3 weight management service. Tier 3 services offer an integrated approach to weight management that is typically delivered by a multidisciplinary team in a hospital setting. At these specific services, patients meet with dieticians, endocrinologists, psychologists, nurse specialists, and social workers on average every other month.

The mechanisms that cause and maintain obesity are extensive, incorporating political, environmental, and societal factors. Encouraging individual dietary behavior change is a major aim of clinics, and patients are seen by a dietician who provides guidance on food choices and nutritional balance. FoodT is an app that delivers food response inhibition training, which aims to reduce the appeal of energy-dense snack foods and the automatic approach responses to cues for such foods [1]. Such training has resulted in a reduction in energy intake and weight loss in adults, which, importantly, was maintained at the 6-month follow-up in a study by Lawrence et al [2]. In another study, a greater number of healthy snack foods were selected by children following such training [3], but to date, FoodT has not been trialed to support children’s snack food choices in a pediatric weight management context. In our trial, it was hoped that when delivered as an adjunctive treatment alongside the extensive social, medical, psychological, and dietetic interventions already offered at a clinic, the introduction of inhibitory control training may offer patients another tool that supports eating choice. This work sought to trial the acceptability and feasibility of delivering the FoodT app within a tier 3 pediatric setting.

## Methods

### Intervention

FoodT is an app based on a go/no-go paradigm; the app prompts users to press a button as rapidly as possible when images of healthy foods (circled in green) are presented and to withhold their response when images of foods high in fat, salt, or sugar (circled in red) are presented [4]. FoodT was designed by Psychologist NSL at the University of Exeter based on the latest inhibitory control research, and it has been tested in many large-scale trials [5-7]. FoodT is available for download on Android and iOS devices. Mobile phones for this study were made available to ensure equal access.

### Procedure

Parents of children at a tier 3 pediatric weight management service were informed of the intervention via letters. The researcher attended the children’s usual clinic appointments and discussed the intervention with parents and children within the clinic waiting room. The trial exclusion criteria included (1) problems with eyesight that could not be corrected with glasses and prevented the participants from seeing the food images on the phone screen; (2) a poor understanding of spoken or written English; and (3) concurrent participation in a trial investigating a weight loss intervention, so as to not confound results. During the trial, no participants were excluded from participating. Willing children and their parents were consented at the clinic by the primary researcher (JSC). Parents and children were offered the option of completing the pretrial questionnaire at the clinic via a laptop or at home. Despite not being a powered trial, the full battery of tests was included in the questionnaire to test the acceptability of the research process. It was requested that participants play FoodT every day for the first week and once per week for the following 4 weeks before automatically being sent a URL link to complete a posttrial questionnaire.

### Ethics Approval

As this was human subject research, National Health Service (NHS) ethical review and approval was granted by the South Central Berkshire NHS research ethics committee (reference number: 18/SC/0471; Integrated Research Application System project ID: 242624). All children assented to participation, and written consent was provided for the children’s participation by their parents or guardians. Parents and guardians consented for their own participation separately. All data presented were deidentified to protect identity. A £5 (US $5.32) voucher was given for each questionnaire completed as compensation for participants’ time.

### Feasibility Measures

The key outcome measures were recruitment (percentage of parents and children who consented to participate), the frequency of app use (target=10 plays), and retention (percentage of participants who completed the intervention). Families who did not consent were asked if they would share their reason.

### Questionnaire

#### Participant Characteristics

Many of the questions included in the questionnaire had been used in previous research with the FoodT app and mirrored in-app questions that were used to collect data on all app users (ie, those downloading the app from the iOS and Android app stores), so as to allow for comparison. Although this was not a powered trial, the questionnaire was included at this point to assess the acceptability of this mode of data collection. Our hope was that in a future fully powered trial, these data would enable analyses that could determine for whom the app works best. Demographic data included postcodes to determine indices of multiple deprivation (a classification of the relative deprivation of small areas in the United Kingdom, where “1” represents the most deprived areas and “10” represents the least deprived areas), sex, age, height, and weight. Accessibility and acceptability were explored via questions regarding current app usage and perceptions on apps for weight management. Children were asked to report which of the clinic’s eight strategies they were currently using to manage their weight. To capture data on disordered eating, the Loss of Control Over Eating Scale [8] and the Children’s Binge Eating Disorder Scale [9] were included, which are validated psychological tools.

#### Measures of How Training Impacts Eating Behavior

Food liking was measured by using a 100-cm visual analogue scale with the end points “love” and “hate.” Children were asked to select foods that they “frequently experience food cravings for” from the 15 training categories and complete a food frequency questionnaire, along with 2 different forced-choice selection tasks. In total, the battery of tests took around 20 minutes to complete.

## Results

The results of the key outcome measures were as follows: the percentage of parents and children who consented to participate (recruitment) was 35% (22/62), the average number of times FoodT was played (frequency of app use; target=10 plays) was 3.6 (SD 3.8), and the percentage of participants who completed the intervention (retention) was 0% (0/22). Families who did not consent were asked if they would share their reason. Among those approached who declined participation (40/62, 65%), the reasons included participants not being interested in using an app, parents feeling that that the app was not applicable to their child, participants not deeming food or eating to be the root of the problem, parents feeling that their child needed to work on more complex issues and their mindset first, and participants feeling overwhelmed by their appointments with the multidisciplinary team and not wanting to take on another intervention. The recruitment period was cut short due to the COVID-19 pandemic.

Participant characteristics can be seen in Table 1. Although most children (7/8, 88%) were engaged with a high number of weight management strategies, 1 child was not actively engaging with any weight management approaches. Although none of the children met clinical thresholds for binge eating disorder, all children demonstrated such tendencies, and several were not classified as individuals with binge eating disorder due to their engagement with compensatory behaviors, such as purging. The foods included in the app were highly liked, and participants often reported cravings for fast food, fizzy drinks, cheese, fruits, and vegetables. The delivery of the intervention via the app was a suitable approach, considering participants’ access to and current engagement with apps (Table 1).

**Figure 1:**
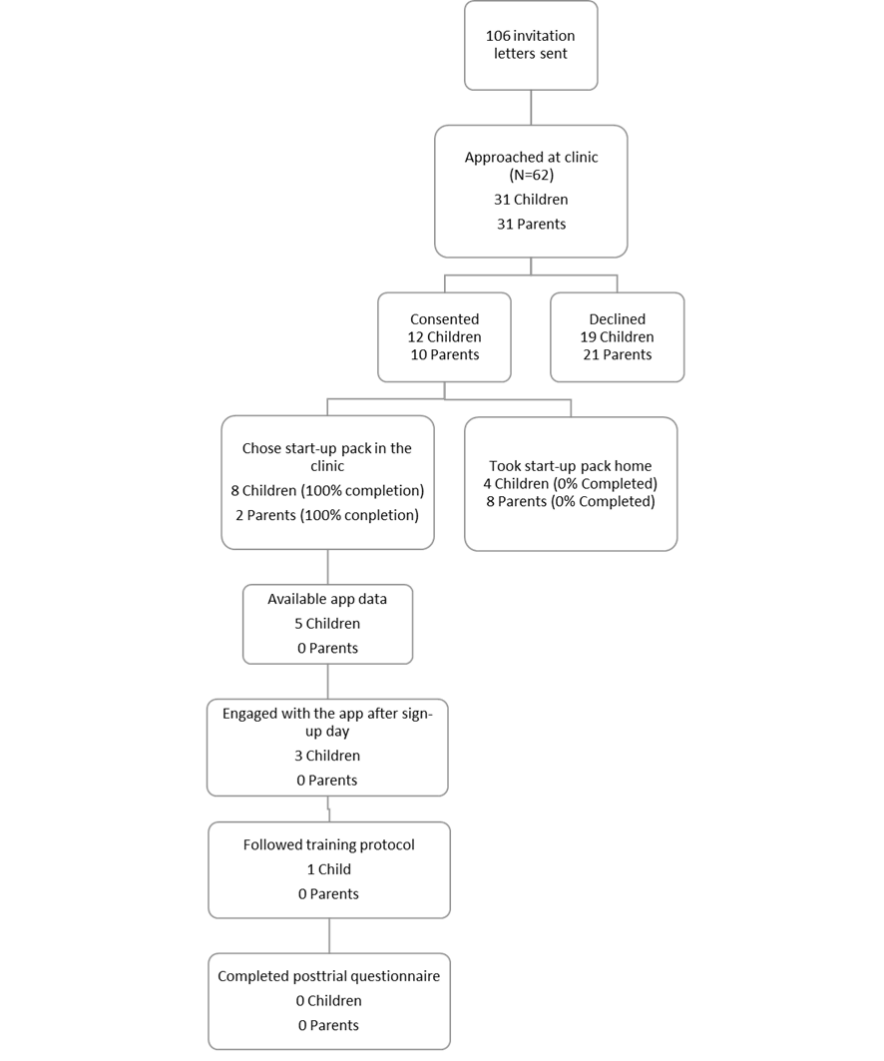
Flow-diagram of recruitment, adherence, and retention.

Of the 8 children who began the trial, app data were available for 5. The remaining participants may not have inputted the code for pairing their data or may not have downloaded the app at all. Of the 5 children, 1 followed the training protocol to meet acceptability thresholds. Further, 2 participants only engaged with the app during the set-up session. None of the children completed the posttrial questionnaires (Figure 1).

**Table 1:** Participant characteristics, eating behavior, and acceptability.

Characteristics					Value
**Child characteristics (n=8)**					
	Age (years), mean (SD)			15.3 (0.95)	
	Female, n			5	
	Weight (kg), mean (SD)			109 (19)	
	Index of multiple deprivation (1=lowest; 10=highest), mean (SD)			3.9 (3.3)	
	Occasionally or often experience loss of control over eating, n			7	
	Presence of clinical binge eating disorder, n			0	
**Child eating behavior (n=8), mean (SD)**					
	Number of weight management strategies being implemented^a^			5 (2.4)	
**Feasibility and acceptability (children: n=8; parents: n=2)**					
	**Recruitment, n (%)**				
		Children (out of the 31 approached)	12 (39)		
		Parents (out of the 31 approached)	10 (32)		
	**App usage (number of plays; target engagement=10 plays), mean (SD)**				
		Children	3.6 (3.8)		
		Parents	0 (0)		
	**Participants with their own smartphone, n**				
		Children	8		
		Parents	2		
	**Time engaged with other apps (hours), mean (SD)**				
		Children	3 (1.3)		
		Parents	—^b^		
	**Retention, n (%)**				
		Children (out of 12 participants)	0 (0)		
		Parents (out of 10 participants)	0 (0)		

^a^The weight management strategies were (1) exercising more, (2) reducing portion size, (3) reducing snacking, (4) eating more fruits and vegetables, (5) eating less foods high in fat, (6) eating less foods high in sugar, (7) changing the timings of their eating, and (8) eating more slowly.

^b^Not available (data not collected).

## Discussion

The primary aim of this work was to explore the acceptability and feasibility of delivering the FoodT app-based intervention within a tier 3 service clinic. We found that this trial was not well accepted, particularly when delivered unsupported via the internet. We will benefit from the outcomes and lessons learned from this feasibility trial, as they are important for planning future work on delivering an app-based intervention in a tier 3 pediatric weight management service.

First, based on the reasons for nonengagement, several parents questioned the suitability of the intervention for their child. This disparity between patient needs and intervention design may be better addressed in future intervention development work that uses a co-design methodology to ensure that the intervention meets the needs of the participants prior to the feasibility trial research stage [10]. A further step toward ensuring that the intervention meets patient needs would be to hold in-depth focus groups with those who declined participation to understand what they would like to see within an app or future intervention. Second, several parents declined participation because they did not feel that their child’s weight was the result of issues with food. This reluctance to accept interventions that target weight via lifestyle changes echo findings from a qualitative study that was conducted at the same clinic [11].

With regard to intervention content, the low recruitment levels could also have been the result of how the intervention was perceived by patients. The situating of the researcher in the public waiting room and the sense that the intervention was not part of the clinic itself may have been barriers to participation. Previous studies conducted in this clinic, which had better recruitment rates, were introduced by trusted clinicians [11].

Importantly, the protocol included many time-intensive measures, which likely acted as a barrier to recruitment and retention. This research was designed to be conducted with minimal researcher involvement to replicate the capacity of the clinical team should the app be rolled out in the future; however, a more supportive environment may be a necessity during this formative period for better establishing the participants’ experiences with the app. Fundamentally, the uncertainty about whether children and parents were deterred from participation due to the concept of FoodT or by the idea of engaging in research limits the conclusions that we can make about the future potential of the app as an inhibitory control training intervention for pediatric weight management. The research team acknowledges that the intervention was weighted toward data collection and would benefit from more patient support and a co-development approach [10].

The research team sought to publish these null results to emphasize the importance of maintaining participant involvement and ensuring that the intervention is right for the population before data collection attempts begin. However, several limitations are acknowledged. The research team recognizes that the mechanisms that cause and maintain obesity are vast and numerous and that the intervention targets 1 potential pathway to supporting individuals. The app was limited in its scope, offering only inhibitory control training, which may have reduced its applicability to all families at the clinic. The weighting toward research processes limited the useful insights into the usability of the app within this setting. Future work could be conducted to test the app alone, without the research package, to better understand its uses.

This work has broad implications, as the findings reflect the importance of prioritizing patients’ needs and ensuring that core outcomes are not lost because of intensive research procedures. In conclusion, little evidence was generated from this research, which leaves many more questions than it answers. In publishing this work, we hope to reduce research waste and ensure that others do not make these costly mistakes.

## Abbreviations

NHS: National Health Service

## Data Availability

The data sets generated and analyzed during this study are available from the corresponding author on reasonable request.
